# Superhydrophobic Coatings Based on PMMA-Siloxane-Silica and Modified Silica Nanoparticles Deposited on AA2024-T3

**DOI:** 10.3390/polym17020195

**Published:** 2025-01-14

**Authors:** Nina Kovač, Barbara Kapun, Matic Može, Iztok Golobič, Slavko Kralj, Ingrid Milošev, Peter Rodič

**Affiliations:** 1 Jožef Stefan Institute, Department of Physical and Organic Chemistry, Jamova c. 39, SI-1000 Ljubljana, Slovenia; nina.kovac@ijs.si (N.K.); barbara.kapun@ijs.si (B.K.); ingrid.milosev@ijs.si (I.M.); 2 Jožef Stefan International Postgraduate School, SI-1000 Ljubljana, Slovenia; 3 Faculty of Mechanical Engineering, University of Ljubljana, Aškerčeva c. 6, SI-1000 Ljubljana, Slovenia; matic.moze@fs.uni-lj.si (M.M.); iztok.golobic@fs.uni-lj.si (I.G.); 4 Jožef Stefan Institute, Department for Materials Synthesis, Jamova c. 39, SI-1000 Ljubljana, Slovenia; slavko.kralj@ijs.si; 5 Faculty of Pharmacy, University of Ljubljana, Aškerčeva c. 7, SI-1000 Ljubljana, Slovenia

**Keywords:** nanoparticles, hybrid sol–gel, adhesion, superhydrophobicity, corrosion

## Abstract

The study aimed to develop a superhydrophobic coating on the aluminium alloy 2024-T3 surface. The desired surface roughness and low surface energy were achieved with SiO_2_ nanoparticles, synthesised via the Stöber method and modified with alkyl silane (AS) or perfluoroalkyl silane (FAS). To enhance particle adhesion to the alloy substrate, nanoparticles were incorporated into a hybrid sol–gel coating composed of tetraethyl orthosilicate, methyl methacrylate, and 3-methacryloxypropyl trimethoxysilane. The coated substrates were characterised using field emission scanning and transmission electron microscopy with energy-dispersive spectroscopy for surface topography, nanoparticle size distribution, composition, and coating thickness. The corrosion resistance of the coatings on AA2024-T3 was evaluated in a 0.1 M NaCl solution using electrochemical impedance spectroscopy. The synthesised SiO_2_ nanoparticles had an average size between 25 and 35 nm. The water contact angles on coated aluminium surfaces reached 135° for SiO_2_ + AS and 151° for SiO_2_ + FAS. SiO_2_ + FAS, indicating superhydrophobic properties, showed the most uniform surface with the most consistent size distribution of the SiO_2_ nanoparticles. Incorporation of nanoparticles into the hybrid sol–gel coating further improved particle adhesion. The ~2 µm-thick coating also demonstrated efficient barrier properties, significantly enhancing corrosion resistance for over two months under the test conditions.

## 1. Introduction

Over the past few years, the development of superhydrophobic coatings with water contact angle (WCA) > 150° and low sliding angle < 10° has been very intensive due to their wide range of applications, such as drug reduction, self-cleaning and anti-icing surfaces, and oil–water separation [1,2]. However, implementing superhydrophobic coatings in industrial or other applications is limited due to various challenges. The main challenge is performing surface durability when the coatings are exposed to harsh conditions, such as stain accumulation and chloride-containing environments [3]. Additionally, combining superhydrophobic properties with other surface functionalities (e.g., antimicrobial effects [4], self-cleaning [5,6,7], anti-icing [5,8,9]) can be challenging in some applications where durability, mechanical, and UV resistance are crucial [7]. Superhydrophobic coatings can be applied on various substrates, for example, textile [10], polymers/glass [11], or metals/their alloys [2,12,13]. Regarding applications of superhydrophobic coatings in corrosion protection, the substrates of interest are aluminium alloys (AA), specifically 2024-T3. This type of wrought aluminium alloy is commonly found in structural and aerospace applications, where material strength and corrosion resistance are critical factors [14]. This limited corrosion resistance is due to the different electrochemical activity resulting from the presence of intermetallics, such as Cu-(Fe, Mn) and Al-Cu-Mg [15,16,17,18,19].

Several techniques were investigated to address the challenges above in creating superhydrophobic surfaces of aluminium and its alloys. The most commonly used techniques are etching [5,20,21,22], laser ablation [8,23,24,25,26], and anodisation [12,27,28]. The shortcomings of these techniques are potential damage to the substrate and inability to deposit on large-scale commercial applications [1,2,7,29]. In contrast, coatings, including low surface energy nanoparticles (NPs), can be coated directly on metals without any effect on the substrates, which makes them more applicable [30,31,32,33]. It has been proved that the durability of the coating depends on two factors: (i) wear-resistant ability of the micro-nano structures and (ii) the adhesion ability of the nanoparticles to the metal substrate [6,34,35,36]. Silica (SiO_2_) NPs prepared by the Stöber method are well-known materials for superhydrophobic coatings due to their abundance, making them the most suitable material for generating nano-level roughness in superhydrophobicity coatings [31,32]. Recently, it was reported that other NPs of different sizes [31,32,37] or types, such as ZnO (WCAs > 160°), retain their properties for up to 1 month of immersion in saline solutions when integrated with polymeric matrices [38,39]. TiO_2_ (WCAs > 155°), with anti-icing and photocatalytic properties [40], and CeO_2_ (WCAs > 158°), retain their properties for several weeks and offer self-healing capabilities [41], and can be used to achieve superior stable superhydrophobic coatings [2,12,13]. However, SiO_2_ particles remain most commonly studied because various surface-functionalising agents can easily modify them, allowing for precise control over hydrophobicity (WCA > 150°) [42,43]. Compared to ZnO and TiO_2_, which may suffer from photocatalytic degradation [38,40], SiO_2_ nanoparticles are more resistant to environmental conditions, ensuring durability. They are also chemically stable and cost-effective, making them ideal for scalable applications [30,32,39]. The Stöber method is also preferred due to the structural compatibility of SiO_2_ with other polymer matrices, ensuring a stable and effective hydrophobic coating for corrosion protection [32,44,45].

The preparation of the wear-resistant superhydrophobic coatings on AA2024-T3 has been reported [39,41], but their durability is often limited by nanoparticle adhesion. To enhance their hydrophobic properties and adhesion, NPs are often modified with organosilane compounds [46] or organically modified molecules (e.g., alkyl or perfluoroalkyl silane) [47]. The difference between modifying SiO_2_ with alkyl silane and perfluoroalkyl silane lies in their chemical structures and the surface energy they impart. Alkyl silane creates a hydrophobic surface by forming a layer of C−H chains, reducing surface energy to 20–30 mN/m, but it cannot repel oils, i.e., it does not exhibit oleophobicity [47]. Perfluoroalkyl silane, with C−F chains, lowers the surface energy to below 10 mN/m, achieving hydrophobicity and oleophobicity [47,48,49]. This makes perfluoroalkyl-modified surfaces ideal for applications requiring extreme water and oil repellence, while alkyl silane is limited to hydrophobic uses, thus achieving only a certain level of improvement.

To further enhance the coating adhesion, different copolymerisation agents can be used, such as polytetrafluoroethylene [31], polymer composite [45], hybrid polymeric nanocomposites [44], hydrophobic agents such as poly(dimethylsiloxane) (PDMS) [50] and hexadecyltrimethoxysilane (HDTMS) [51], with low surface energy. For poly(methyl methacrylate) (PMMA)-siloxane-silica coatings, studies reported on the effect of the addition of lignin [52] and cerium [53,54] in achieving excellent anti-corrosion performance. However, the impact of the addition of SiO_2_ NPs modified with alkyl and perfluoroalkyl silanes to achieve superhydrophobic properties, along with their influence on anti-corrosion performance and mechanical properties, has not been reported.

Synthesising the hybrid sol–gel of the PMMA-siloxanes-silica has good reproducibility and gives high purity and homogeneous coatings [55,56]. Combining the organic polymer (PMMA) [56,57] and the inorganic silica domains produces hybrids with tailored combinations of the toughness of the PMMA polymer and the hardness of the inorganic silica phase [56,58,59,60]. There are several studies of various silica-siloxane PMMA coatings prepared under different synthesis conditions, differing in methyl methacrylate (MMA)/3-methacryloxypropyl trimethoxysilane (MAPTMS) ratio [56] and the ratio of water/ethanol in the inorganic phase [56,61,62,63,64,65,66]. In the past, PMMA coatings have been deposited on several surfaces and used as barrier corrosion protection for aluminium alloys [62,64,66] and other metals such as steel [56,67]. Recent studies of the PMMA-silica system have shown that PMMA chains in the structure are covalently cross-linked and can also be strongly adhered to the substrate [65] due to the covalent bonds formed between the hybrid and hydroxide species silanol groups at the coating/substrate interface [62,65,66].

This work uniquely integrates modified SiO_2_ nanoparticles (functionalised with alkyl and perfluoroalkyl silanes). In addition, it is the first time the effective integration of modified SiO_2_ nanoparticles into a PMMA-siloxane-silica has been demonstrated. This approach aims to achieve the superhydrophobic character of the AA2024-T3 and improve the adhesion between modified SiO_2_ NPs and the aluminium surface through PMMA siloxane silica coating (abbreviated as TMM). Enhanced surface roughness, superhydrophobicity, and superior (prolonged) corrosion resistance are the main advantages compared to those studied previously.

## 2. Materials and Methods

### 2.1. Chemicals and Material

**Figure 1:**
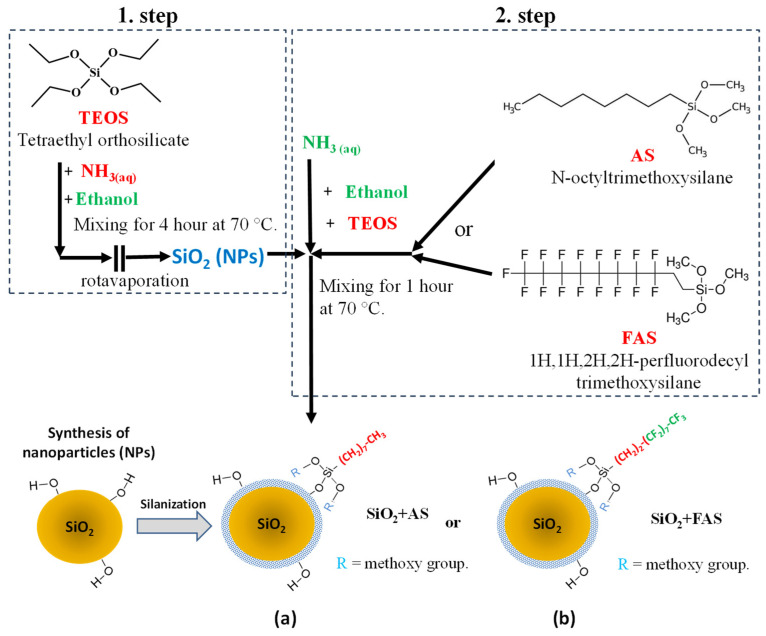
Flowchart depicting silica nanoparticles’ synthesis and surface modification following Stöber’s one-step process. The synthesis begins with the hydrolysis and condensation of tetraethyl orthosilicate (TEOS) in the presence of ammonia (NH_3_) and ethanol. Subsequently, the surface of the synthesised SiO_2_ nanoparticles was modified using two silane reagents: alkylsilane (AS) and perfluoroalkyl silane (FAS). The modifications yielded two types of NPs: (**a**) SiO_2_ + AS, featuring hydrophobic alkyl-functionalised surfaces, and (**b**) SiO_2_ + FAS. The silanisation reaction produces covalently bonded SiO_2_ nanoparticles with hydrophobic chains oriented outward. During this process, water (H_2_O) and alcohol (e.g., ethanol or methanol) are generated as side-products due to hydrolysis and condensation reactions.

Chemicals with high purity were used to achieve controlled synthesis of silica SiO_2_ nanoparticles and their surface functionalisation. Tetraethyl orthosilicate (TEOS, ≥99.0%, Sigma-Aldrich, St. Louis, MI, USA) served as a silica precursor, while an ammonia solution (25%, Emsure) catalysed the hydrolysis and condensation steps in the presence of NH_3_ and 2-propanol (≥99.8%, Honeywell, Charlotte, NC, USA) as a solvent in the sol–gel process. N-octyltrimethoxysilane (95%, Fluorochem, Glossop, UK) (AS) and 1H,1H,2H,2H-perfluorooctyltrimethoxysilane (FAS) (95%, Fluorochem) were employed as silane agents to modify the surface of the SiO_2_ nanoparticles, enhancing their hydrophobicity, as presented in Figure 1.

Methyl methacrylate (MMA, 99%, Sigma-Aldrich) and 3-methacryloxypropyl trimethoxysilane (MAPTMS, 98%, Sigma-Aldrich) were used as monomers for the copolymerisation reactions, facilitated by benzoyl peroxide (BPO) (with 25% H_2_O, Merck Millipore), which served as a free radical initiator. Tetrahydrofuran (THF, ≥99.9%, Sigma-Aldrich) and ethanol (absolute, for analysis, Sigma-Aldrich) were used as organic solvents to dissolve and dilute in certain stages of the reaction.

Aluminium alloy (AA2024-T3) with dimensions of 40 mm × 15 mm × 0.5 mm was used for coating deposition. It was distributed by Kaiser Aluminium, Lake Forest, CA, USA. Its standard composition in weight percentage (wt.%) consists of 3.8–4.9 wt.% copper (Cu), 1.2–1.8 wt.% magnesium (Mg), 0.50 wt.% silicon (Si), 0.50 wt.% iron (Fe), 0.3–0.9 wt.% manganese (Mn), 0.25 wt.% zinc (Zn), and 0.4 wt.% other trace metals.

### 2.2. Sample Preparation

AA2024-T3 surface preparation involves multi-step mechanical grinding using a grinding/polishing machine (LaboPol-20 system, Struers, Cleveland, OH, USA). The surface was ground sequentially with 1200, 2400, and 4000 grit silicon carbide abrasive papers (Struers, Cleveland, OH, USA) to achieve a smooth and uniform appearance without visible grinding tracks. This process was designed to remove the spontaneously formed oxide film. Following grinding, the samples underwent ultrasonic cleaning in ethanol for 5 min using an ultrasonic cleaner (Elmasonic S10H, Elma Schmidbauer GmbH, Singen, Germany) to remove grinding residuals. Finally, the ground samples were thoroughly rinsed with pure ethanol and dried with a compressed nitrogen stream to ensure a contaminant-free surface before coating deposition.

### 2.3. Synthesis and Deposition of the Coating

#### 2.3.1. Synthesis of the Nanoparticles and Their Modification

The synthesis of SiO_2_ NPs was performed following established protocols as outlined in previous studies [37,68]. This manuscript focuses on the detailed characterisation of the synthesised products. The SiO_2_ NPs were synthesised by a two-stage (batch) hydrolysis reaction of TEOS (serves as the silica precursor to form the SiO_2_) and surface modification with organically modified silane AS and FAS containing an alkyl (C–H) or perfluoro (C–F) chain.

In the first step, 3 mL of NH_3(aq)_ (acts as a catalyst and, due to the presence of water, initiates and promotes the hydrolysis of TEOS) was mixed with 50 mL of ethanol (used as a solvent, providing a medium for the uniform dispersion of TEOS and ensuring controlled nanoparticle growth) and stirred at 60 °C for 30 min (Figure 1). Then, 3 mL of TEOS was added dropwise into the mixture and stirred for 1 h [68]. The final solution was evaporated using the laboratory rotary vacuum evaporator (Chongqing Drawell Instrument Co., Ltd., Chongqing, China) to remove the solvent and obtain the SiO_2_ nanoparticles powder.

In the second step, the surface modification of the prepared nanoparticle powder was performed by following the procedure. SiO_2_ NPs were ultrasonically dispersed in a mixture of ethanol and isopropanol (1:1, *v*/*v*) at a concentration of 7.5 mg/mL at room temperature using Elmasonic S 40 ultrasound for 20 min [37]. Then, the AS (0.5 wt.%), TEOS (0.1 wt.%), and NH_3_(aq) (20 wt.%) were added into the suspension, respectively (Figure 1a). Finally, they were stirred for 4h to hydrolyse TEOS and AS (introduces alkyl chains to the SiO_2_ surface, increasing hydrophobicity) completely, forming SiO_2_ + AS through condensation Reaction (1).

particle-Si−OH + HO−Si(OR)_2_-(alkyl or perfluoroalkyl chain) → particle-Si−O−Si(OR)_2_-(alkyl or perfluoroalkyl chain) + H_2_O (R1)

A similar reaction is also assumed for (SiO_2_ + FAS) where instead of AS, perfluoro silanes (FAS) were used (introduces perfluoroalkyl chains to the SiO_2_), as shown in Figure 1b, Reaction (1).

As presented in Figure 1a,b, the AS and FAS molecules were covalently bonded on the nanoparticle surface, forming Si–O–Si bonds. The side product of the reaction is also methanol. The alkyl and perfluoroalkyl chains are oriented outside.

#### 2.3.2. Synthesis of the Hybrid Sol–Gel Coating

**Figure 2:**
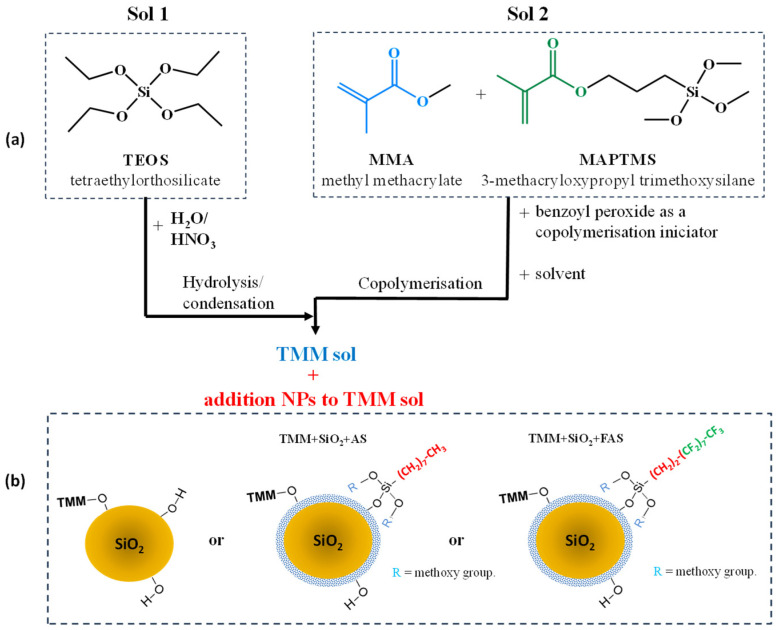
(**a**) Flow chart of the preparation of the PMMA-siloxane-silica TMM sol by combining Sol 1 and Sol 2 and (**b**) network formation of TMM sol with added NPs.

Organic–inorganic (hybrid) sol–gel solutions were prepared as two separately synthesised sols: (i) Sol 1: hydrolysed/condensed TEOS (silica sol) and (ii) Sol 2: copolymerised MMA/MAPTMS sol in THF (used as a solvent to ensure uniformity of formed products). The molar ratio of all used reagents was: TEOS:EtOH:H_2_O:MMA:MAPTMS:BPO:THF = 2:5.3:10.5:8:1:0.08:53 [62,66,69,70]. Acidified water (H_2_O/H^+^) prepared from nitric acid HNO_3_ had a pH = 1. Sol 1 was prepared during the dropwise addition of ethanol and H_2_O/H^+^ into TEOS. Then, the solution was mixed for 30 min at 23 °C using the magnetic stirring bar. The radical copolymerisation between MMA and MAPTMS was performed in the presence of BPO. The solution was mixed vigorously for 4 h at 70 °C (under reflux). After this, the Sol 2 was cooled down to ambient temperature and Sol 1 was added dropwise. The prepared solution was mixed for 1h at ambient temperature to obtain the final TMM sol, Figure 2.

#### 2.3.3. Coatings Deposition

Coatings were prepared using the following protocols:

(a) The NPs dispersed in tetrahydrofuran (THF) to ensure their uniform distribution. Then, the solution was sprayed directly on the AA2024-T3 surface from a distance of 10 cm using hand spray;
(b) The TMM solution was deposited to the AA2024-T3 surface using a dip-coater. The alloy sheets were attached to the dip-coater holder and dipped into a glass beaker with the TMM solution for 3 s. The dip-in and pull-out rates were 14 mm/min. The coating was deposited on the alloy surface only once;
(c) Protocol (b) was followed by hand spraying modified silica nanoparticles dispersed and 5 times diluted (with THF) onto TMM coating.

After each deposition method, coated AA2024-T3 samples were thermally cured at 180 °C for 1 h to achieve a final (dry) coating. Prepared samples were used for further characterisation.

### 2.4. Synthesis Coating Characterisation

#### 2.4.1. Scanning Electron Microscope

The morphology of the SiO_2_ NPs, PMMA-siloxane silica sol–gel coatings and coatings with incorporated SiO_2_ NPs deposited on AA2024-T3 was analysed using a scanning electron microscope (SEM) FEI HeliosNanolab 650, which is coupled with an energy dispersive X-ray spectrometer (EDS) (Oxford Instruments X-max SDD (50 mm^2^) detector). The spectra were collected and analysed using AZtec software (version 6.1). The SEM micrographs were recorded using the secondary electron imaging mode at 5 kV energy. Accompanying analysis by EDS was conducted at 10 kV beam voltage. Before analysis, the coating on the AA2024-T3 surface was scribed using the diamond blade, along which the coating thickness was determined.

#### 2.4.2. Transmission Electron Microscope

The morphology of the SiO_2_ NPs was analysed using a transmission electron microscope (TEM). The micrographs were acquired on JEM 2100 (Jeol, Tokyo, Japan) at an accelerating voltage of 200 kV. Samples for TEM analysis were prepared by dispersing the synthesised and functionalised silica nanoparticles in THF using ultrasonication for 2 min to ensure uniform dispersion. A drop (~20 μL) of the nanoparticle suspension was then deposited onto a carbon-coated copper grid using a micropipette. The grid was allowed to air-dry under ambient conditions for 1 h before analysis.

#### 2.4.3. Water Contact Angle Measurements

Water contact angle measurements were performed at ambient temperature using a Krüss FM40 EasyDrop contact-angle measuring system. Measurements were performed according to standard (ASTM-D7334-08-2022) [71] with the static sessile-drop method. These measurements were performed on the PMMA-siloxane silica sol–gel coatings with incorporated modified SiO_2_ NPs and on sprayed samples with modified SiO_2_ NPs. A water droplet (6 μL) was formed on the end of the syringe. Then, the droplet was carefully deposited onto the tested surface. Digital images of the droplet silhouette were captured a few seconds after droplet deposition using a high-resolution camera, and the contact angle was determined by numerically fitting the droplet image using the software for drop shape analysis. The values reported herein present the average of at least three measurements on randomly chosen areas.

#### 2.4.4. Measuring Corrosion Properties

The corrosion (protective) performance of uncoated and coated AA2024-T3 with prepared coatings was tested under immersion in corrosive medium at 25 ± 2 °C in 0.1 M NaCl solution to simulate a corrosive environment commonly encountered in industrial and everyday applications and ensure reproducibility and comparability of results with existing literature. The solution was prepared using Milli-Q Direct water with a resistivity of 18.2 MΩ·cm^2^ at 25 °C (Millipore, Billerica, MA, USA). Electrochemical measurements were carried out in a standard three-electrode corrosion cell. The surface area of aluminium exposed to the corroding solution (served as a working electrode) was 1 cm^2^. A silver/silver chloride (Ag/AgCl, sat. KCl, *E* = 0.197 V vs. saturated hydrogen electrode) was used as the reference electrode. The carbon rod served as a counter electrode. Electrochemical experiments were carried out with an Autolab PGSTAT204M (Metrohm Autolab, Utrecht, The Netherlands) potentiostat/galvanostat and controlled by Nova 2.1.7 software. Electrochemical impedance spectra (EIS) were acquired in the frequency range from 100 kHz to 10 mHz with a 10 mV amplitude (rms) signal. The reproducibility of the EIS experiments was confirmed by performing three independent measurements for each coating type. The representative curves after selected immersion times (1 day, 1 month and 2 months) are plotted in the Figures.

#### 2.4.5. Adhesion Testing

The X-cross tester kit was used to test the adhesion of coated samples with TMM + SiO_2_ NPs. The surface was cleaned of any surface impurities, and the scribes on the sample surface with the intersection in the middle were made with a specific tool (diamond razors). The patterned surface was covered with #810 ScotchMagicTM tape. Then, the tape was pressed to the surface, and slowly peeled off. The degree of adhesion was assessed based on the amount of coating fragments removed from the patterned surface.

## 3. Results

### 3.1. Synthesis of Silica Nanoparticles

**Figure 3:**
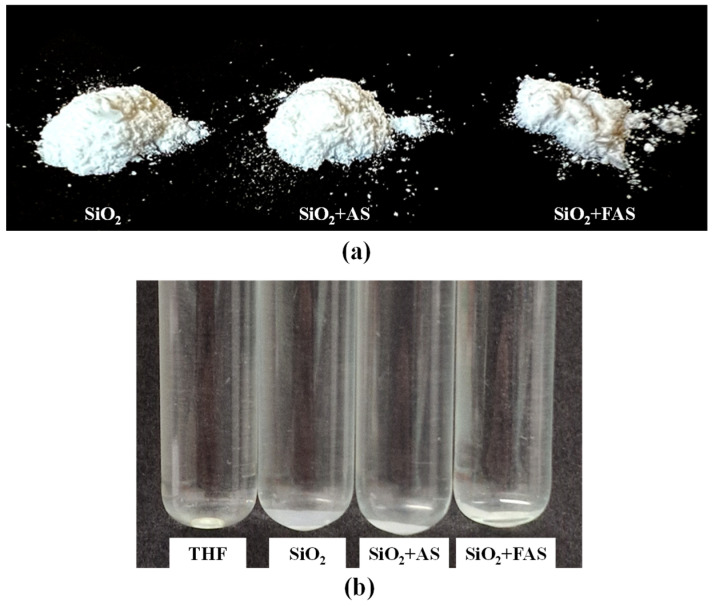
(**a**) The appearance of the SiO_2_, SiO_2_ + AS, and SiO_2_ + FAS NPs in powder form and (**b**) their THF suspensions.

Figure 3a illustrates the appearance of the synthesised NPs: unmodified SiO_2_, SiO_2_ modified with n-octyltrimethoxysilane (SiO_2_ + AS), and SiO_2_ modified with perfluorooctyl silane (SiO_2_ + FAS). All synthesised nanoparticles appeared as a fine, white powder, although some textures varied with the type of surface modification. Specifically, the SiO_2_ + FAS powder exhibited a finer and more cohesive structure, likely due to the hydrophobic properties introduced by the perfluorooctyl silane modification, which alters the surface characteristics and affects particle agglomeration during synthesis.

Figure 3b shows the dispersion behaviour of these three types of NPs in THF. Dispersed nanoparticles form a slightly turbid suspension, indicated by adding NPs to the THF solution. This similar turbidity of the suspension suggests that the hydrophobic and hydrophilic properties of the different surface modifications do not impact interaction with THF and form colloidal stability.

#### 3.1.1. Surface Analysis of Synthesised SiO_2_, SiO_2_ + AS, and SiO_2_ + FAS

As given in the supplement data, the synthesised SiO_2_ NPs using the Stöber method were analysed using FTIR (Figures S1 and S2) and transmission electron microscopy (TEM) (Figure S3) to evaluate their surface modification, size and morphology.

The FTIR spectra confirmed the bonded AS and FAS on SiO_2_ NPs, which were seen as the formation of the Si–O–Si bonds and C–H and C−F bands related to the alkyl and perfluoroalkyl tail in the molecule.

Selected area electron diffraction (SAED) analysis confirmed the amorphous structure of the SiO_2_ nanoparticles. This aligns with the Stöber synthesis process, where amorphous structure is expected.

**Figure 4:**
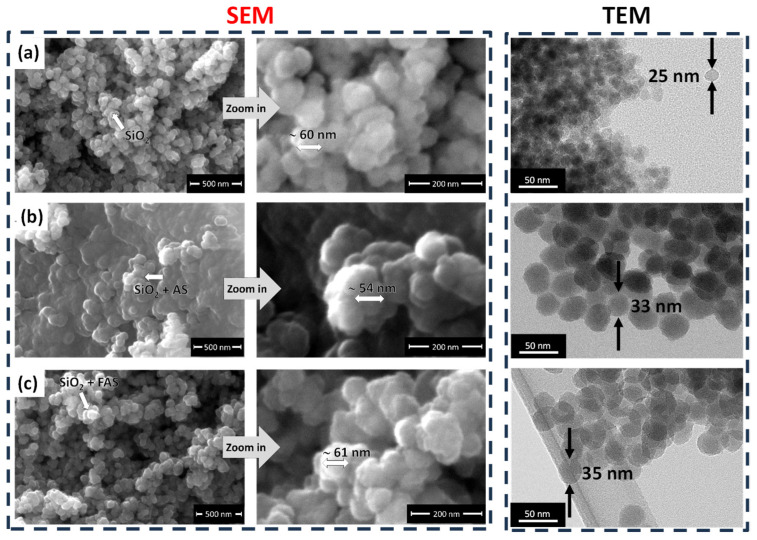
Secondary electron (SE) SEM images at different magnifications (50,000× and 200,000×) of (**a**) SiO_2_, (**b**) SiO_2_ + AS, and (**c**) SiO_2_ + FAS nanoparticles and corresponding TEM micrographs (right side images).

Figure 4 presents representative secondary electron (SE) SEM images (left panel) and TEM images (right panel) of unmodified SiO_2_ NPs, SiO_2_ + AS NPs, and SiO_2_ + FAS NPs, captured at both middle (50,000×) and high magnifications (200,000×). These images highlight the morphology, size distribution, and surface characteristics of the synthesised NPs.

The unmodified SiO_2_ NPs (Figure 4a) exhibit a nearly monodispersed and spherical morphology, with a high degree of homogeneity across the imaging of the sample. The average diameter obtained by TEM was ~25 nm (and SEM was ~60 nm). A slightly greater size of SiO_2_ obtained with SEM compared to TEM is due to a lower resolution of SEM and the fact that the NPs were carbon-coated before SEM imaging. EDS analysis confirmed the elemental composition of Si and O, with atomic percentages of 32.3% and 67.7%, respectively (Table 1), aligning with the expected 1:2 atomic ratio of SiO_2_.

The SiO_2_ + AS NPs (Figure 4b) show similar particle size, with an average diameter of approximately ~33 nm (~54 nm obtained by SEM). SiO_2_ + AS NPs exhibit a slightly increased size compared to unmodified NPs due to forming an organic shell of n-octyltrimethoxysilane on the SiO_2_ surface. The SEM image indicated less dispersed particles and some larger agglomerates (Figure 4b). The shell formation suggests that the AS coating slightly alters the surface properties of the NPs, promoting mild aggregation while maintaining a generally spherical morphology. The EDS data stated a similar composition as unmodified SiO_2_ because the presence of AS cannot be distinguished with EDS (Si is already present in SiO_2_, and C was omitted from the EDS analysis since the samples were carbon-sputtered before the analysis).

The SiO_2_ + FAS NPs (Figure 4c) retain a monodispersed, spherical structure with the average particle size of 35 nm (with SEM ~61 nm), consistent with the unmodified SiO_2_. This stability in size suggests that the perfluorooctyl silane (FAS) coating forms a thin, well-defined shell on the NP surface without significantly affecting its overall dimensions. A notable F content (13.0 at.%) in the EDS analysis confirms the successful incorporation of FAS onto the SiO_2_ surface, imparting a hydrophobic character to the nanoparticles. The stable, monodispersed nature of SiO_2_ + FAS indicates that the FAS layer is well-bonded but delicate, as it does not significantly increase particle size. The better distribution of the particles is probably due to the presence of the perfluoro chain and interactions between the chains [72,73], which offer more even coverage of NPs and prevent their agglomeration compared to SiO_2_ + AS NPs.

In Table 1, EDS data confirms the elemental composition of the unmodified SiO_2_ and verifies the successful surface functionalisation of SiO_2_ with AS and FAS, as indicated by the observed changes in composition, particularly the significant presence of F (13 at.%) in the SiO_2_ + FAS sample. These surface modifications can be further correlated with dispersibility and surface energy.

#### 3.1.2. The Surface Appearance of SiO_2_, SiO_2_ + AS, and SiO_2_ + FAS Deposited on the AA2024-T3 Surface

**Figure 5:**
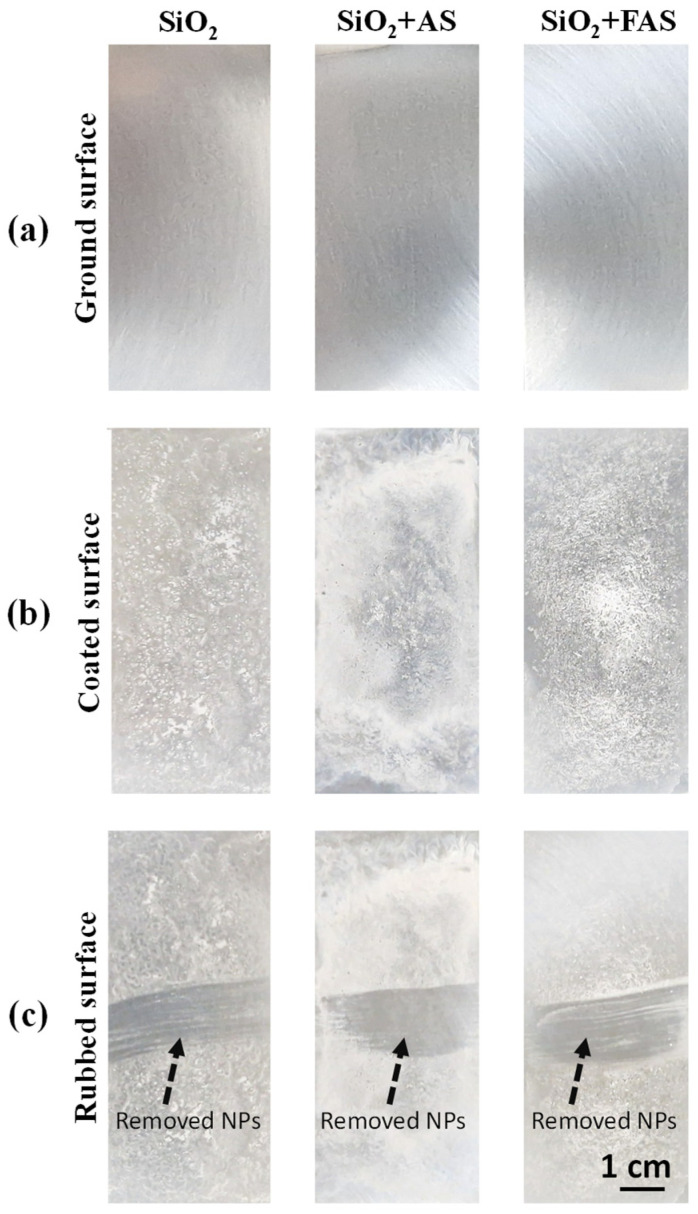
Surface appearance of (**a**) ground AA2024-T3 and (**b**) SiO_2_ NPs, SiO_2_ + AS, and SiO_2_ + FAS deposited on the AA2024-T3 surface. (**c**) Surface appearance after rubbing the surface.

A thin film was formed by spraying the THF suspension of NPs onto AA2024-T3. Figure 5 shows the surface appearance of each type of NP film: unmodified SiO_2_, SiO_2_ + AS, and SiO_2_ + FAS. The results indicate that unmodified SiO_2_ and SiO_2_ + AS coatings exhibited visible agglomerates and uneven distribution, as shown in Figure 5. The images reveal distinct differences in surface uniformity across the samples. The SiO_2_ and SiO_2_ + AS films show visible agglomerates, indicating that the NPs did not distribute evenly across the surface. This uneven distribution suggests that the unmodified and AS-modified nanoparticles exhibit limited dispersion stability in THF, resulting in a less uniform coating upon drying. This is probably related to particle agglomeration, presented in SEM images (Figure 4b).

In contrast, SiO_2_ + FAS displayed a significantly more homogeneous and evenly distributed coating, attributed to the enhanced dispersion stability of the perfluoro alkyl chain of FAS molecules. Despite this improvement, isolated non-uniformed regions were still observed, allowing better particle distribution and a smoother film with improved adhesion, likely due to the hydrophobic nature of the perfluorooctyl silane layer.

#### 3.1.3. Water Contact Angles of SiO_2_, SiO_2_ + AS and SiO_2_ + FAS Deposited on AA2024-T3

**Figure 6:**
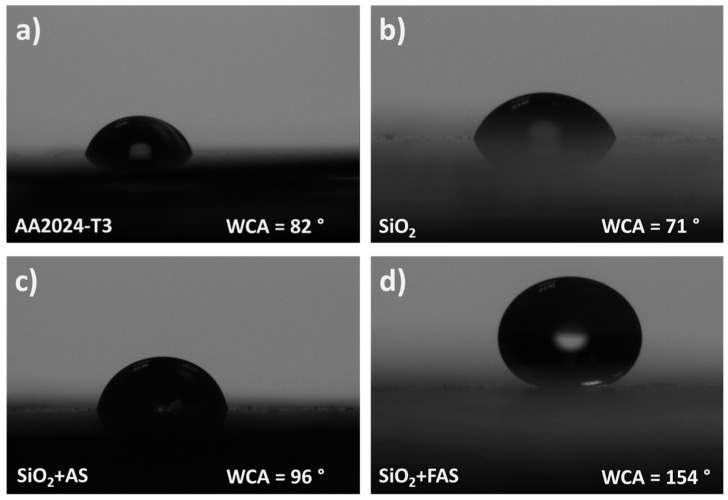
Water droplets on the (**a**) AA2024-T3 substrate and the modified substrate using (**b**) SiO_2_, (**c**) SiO_2_ + AS, and (**d**) SiO_2_ + FAS NPs.

To evaluate the effect of different surface modifications on the wettability of SiO_2_-coated AA2024-T3 surfaces, water contact angle (WCA) measurements were conducted, Table S1. Figure 6 illustrates the changes in water droplet shapes and WCA values for AA2024-T3 coated with unmodified SiO_2_, SiO_2_ + AS, and SiO_2_ + FAS, highlighting the influence of each type of surface treatment.

The uncoated AA2024-T3 surface (Figure 6a) exhibited a low WCA of 82°, where the water droplet spread significantly, forming a near-circular droplet. The low WCA reflects the inherent hydrophilic nature of the AA2024-T3 surface, which readily interacts with water.

Upon coating with unmodified SiO_2_ NPs (Figure 6b), the WCA decreased to 71°, showing a similar, near-circular droplet shape. However, the surface remains relatively hydrophilic without additional chemical modification.

The SiO_2_ + AS coating (Figure 6c) displayed a higher WCA of 96°, indicating enhanced water-repellence compared to unmodified SiO_2_. This increase is attributed to the alkyl chain of n-octyltrimethoxysilane, which reduces surface energy and improves hydrophobicity. The shape of the water droplet became more spherical, further demonstrating the increased water-repellent properties introduced by AS modification.

The most significant effect was observed with the SiO_2_ + FAS coating (Figure 6d), which showed a WCA of 154°, characteristic of a superhydrophobic surface. The water droplet formed an almost perfect sphere, demonstrating minimal interaction with the surface. The superhydrophobicity is due to (i) the FAS modification, as the perfluoro alkyl chains drastically reduce surface energy, much more effectively than the AS-modified surface [49,72], and (ii) better distribution of fine NPs on the surface (less agglomeration). These results suggest that surface modification plays a crucial role in dictating the wettability of SiO_2_-coated surfaces, with the FAS modification imparting markedly lower surface energy than the alkyl chain modification in SiO_2_ + AS.

WCA measurements confirmed that although increased surface roughness of SiO_2_ NPs contributes to higher WCA values, the chemical nature of the surface modification is the primary determinant of hydrophobicity, with FAS achieving superhydrophobic properties due to its low surface energy.

#### 3.1.4. Adhesion of the NPs on the AA2024-T3 Surface

Figure 5c presents the surface appearance of AA2024-T3 coated with unmodified SiO_2_, SiO_2_ + AS, and SiO_2_ + FAS after undergoing an adhesion test. NP coatings on all three samples exhibit poor adhesion to the AA2024-T3 substrate. In the case of unmodified SiO_2_, the coating appears irregular and visibly peeled in certain areas, highlighting significant detachment and weak bonding to the alloy. Similarly, the film was partially detached on the SiO_2_ + AS sample, reflecting weak adhesion.

The SiO_2_ + FAS coating, while demonstrating an initially smooth and even distribution, showed signs of detachment after the adhesion test. Despite the enhanced dispersion and uniformity in the initial deposition, the FAS-modified layer similarly lacks sufficient bonding strength to the AA2024-T3 surface.

These findings confirmed that while surface modifications can alter wettability and distribution properties, they do not necessarily improve the adhesion of the NP coatings at the AA2024-T3 surface. The poor adhesion across all samples suggests that additional surface treatments or adhesion-promoting agents are required to enhance the durability and stability of SiO_2_-based coatings on AA2024-T3 surfaces [31,44,45,50]. The solution to this problem was approached by using hybrid sol–gel coatings for NP incorporation.

### 3.2. Synthesis of Hybrid Sol–Gel Coating

**Figure 7:**
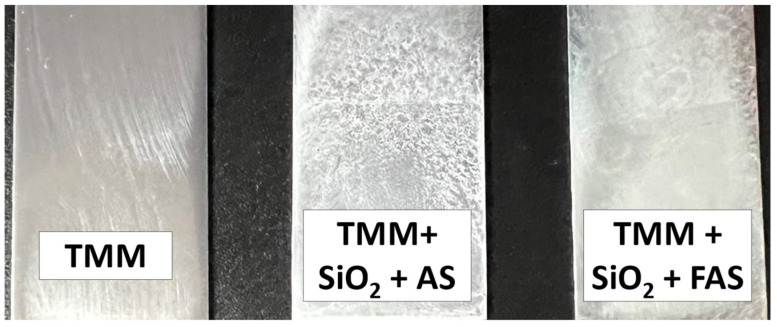
The surface appearance of the TMM, TMM + SiO_2_ + AS, and TMM + SiO_2_ + FAS coating deposited on AA2024-T3.

Sols were synthesised following a structured three-step process to prepare the coatings with selected properties. The schematic illustration of synthesis is given in Figure 2a. Figure 7 displays the surface appearance of AA2024-T3 aluminium alloy samples coated with different TMM-based formulations: unmodified TMM, TMM with SiO_2_ modified by AS (TMM + SiO_2_ + AS), and TMM with SiO_2_ modified by FAS (TMM + SiO_2_ + FAS). The unmodified TMM coating appears almost transparent on the surface, suggesting good optical transparency and even distribution. TMM + SiO_2_ + AS coating reflects white due to SiO_2_ NPs, which scatter light and introduce a visible texture to the coating. However, the nanoparticles’ distribution appears less uniform than the FAS-modified sample. Adding SiO_2_ + FAS also results in a white-coloured coating with a noticeably more uniform appearance than the SiO_2_ + AS. The improved distribution of SiO_2_ NPs in the FAS-modified coating could be attributed to the hydrophobic characteristics imparted by the perfluoro alkyl chain, enhancing the dispersion and stability of nanoparticles within the TMM matrix.

These observations indicate that including surface-modified SiO_2_ nanoparticles significantly affects the visual appearance and particle distribution within the TMM coating, with FAS modification promoting a more homogeneous and stable film.

#### 3.2.1. Surface Characterisation of the Coating

**Figure 8:**
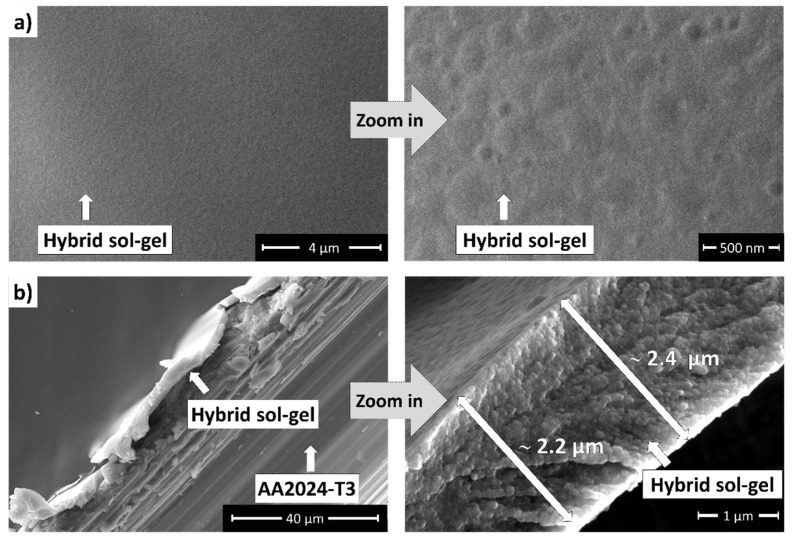
(**a**) Top-view SEM images of the TMM coating deposited on AA2024-T3 at low (**left**) and high (**right**) magnification. (**b**) SEM image along the artificially performed coating cross-section with the estimated thickness of ~2.3 μm.

The TMM coating and its modifications with SiO_2_ NPs were analysed using SE SEM imaging to evaluate surface morphology, coverage, and thickness, as shown in Figure 8.

The unmodified TMM coating, shown in Figure 8a, displays a smooth and uniform surface free from visible cracks, flaking, or pores. Along an artificial scribe (Figure 8b), the coating thickness is estimated to be ~2.3 μm, aligning with thicknesses observed in similar siloxane-silica coatings [52,56,64,66,69,70,74].

**Figure 9:**
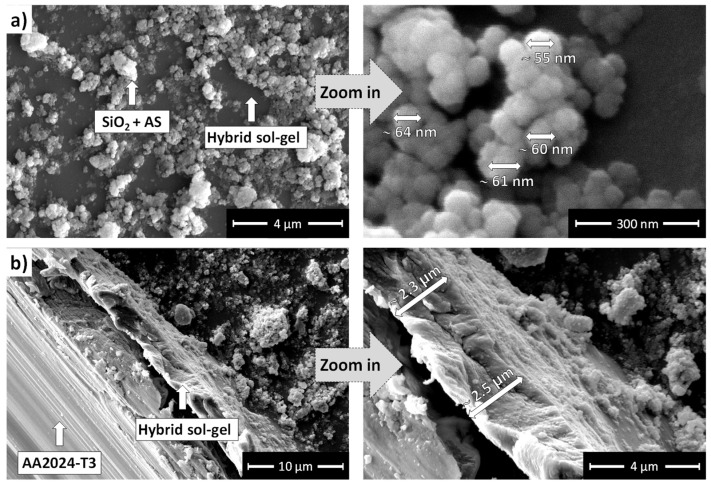
(**a**) Top-view SEM images of the TMM + SiO_2_ + AS coating deposited on AA2024-T3 at low (**left**) and high (**right**) magnification. (**b**) SEM image along the artificially performed coating cross-section with the estimated thickness of ~2.4 μm.

The SEM micrographs of the TMM + SiO_2_ + AS coating are presented in Figure 9. The surface morphology reveals a rough texture due to the SiO_2_ + AS particles. The NPs are not uniformly distributed, leading to the formation of noticeable agglomerates. Despite this uneven dispersion, the underlying TMM coating layer remains smooth and intact, free from visible cracks. However, certain surface regions appear uncovered. A higher magnification image demonstrates that the individual SiO_2_ NPs have diameters ranging from approximately 55 nm to 64 nm (Figure 4b).

Figure 9b presents the cross-sectional SEM view along a scribed coating region. The TMM + SiO_2_ + AS coating exhibits a uniform structure with a similar thickness of ~2.4 μm compared to standard TMM coating (Figure 8). The interface between the coating and the AA2024-T3 is smooth, with no evidence of delamination or cracking, ensuring the structural integrity of the system.

**Figure 10:**
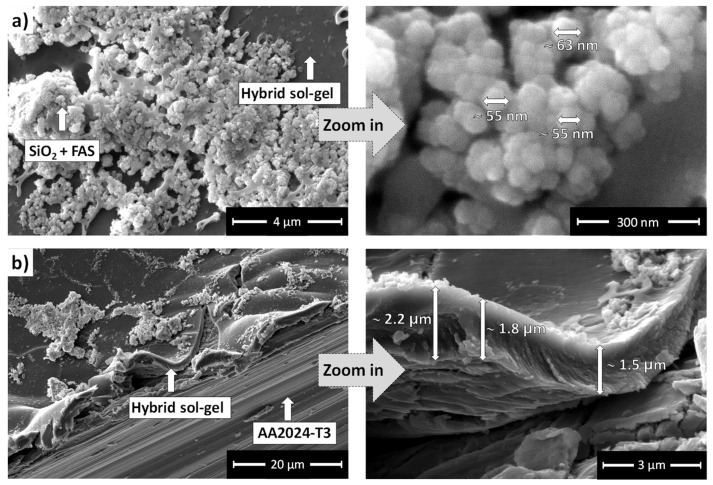
(**a**) Top-view SEM images of the TMM + SiO_2_ + FAS coating deposited on AA2024-T3 at low (**left**) and high (**right**) magnification. (**b**) SEM image along the artificially performed coating cross-section with estimated thickness of ~1.8 μm.

The SEM image of the TMM + SiO_2_ + FAS coating is shown in Figure 10a. It has a rough surface similar to the TMM + SiO_2_ + AS, with evenly distributed SiO_2_ agglomerates. As with the AS-modified version, the base layer of the coating remains smooth and intact without visible cracks, though some surface areas are uncovered.

The cross-sectional SEM image along the scribe (Figure 10b) reveals that the TMM + SiO_2_ + FAS coating is slightly thinner than the other two coatings, with an estimated thickness of ~1.8 μm. Although this thickness is marginally lower, it is within the expected experimental error for coating deposition and measurement, possibly caused by the differences in the deposition process or nanoparticle adhesion and distribution within the TMM matrix.

The surface morphology and thickness measurements reveal that deposition of SiO_2_ + AS on TMM coating enhances surface roughness, but the overall coating thickness remains within a similar range across all samples. The TMM + SiO_2_ + AS and TMM + SiO_2_ + FAS coatings show more textured surfaces with nanoparticle agglomerates, providing added surface features without significantly altering the coating’s overall thickness.

#### 3.2.2. Water Contact Angles of the Coated Aluminium Alloy AA2024-T3

**Figure 11:**
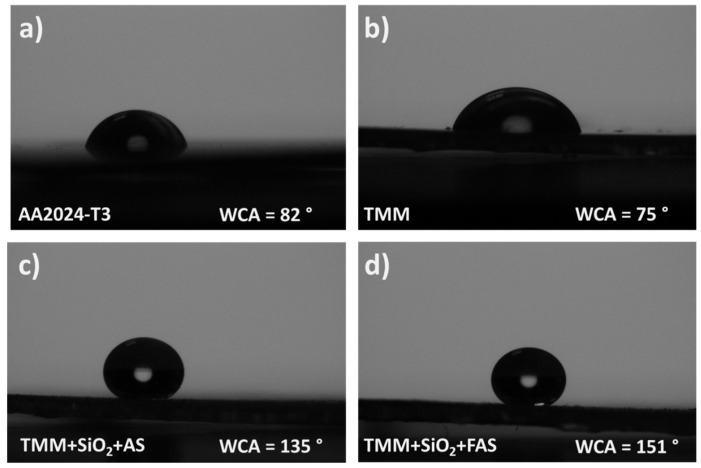
Schematic presentation of a water droplet on a solid surface of (**a**) ground AA2024-T3 and coated with (**b**) TMM, (**c**) TMM + SiO_2_ + AS, and (**d**) TMM + SiO_2_ + FAS coating.

The wettability of AA2024-T3 surfaces coated with various TMM-based formulations was evaluated through WCA measurements, as shown in Figure 11. The uncoated alloy exhibited a WCA of 82°, resulting in a half-circular droplet shape. This level of hydrophilicity is commonly attributed to the naturally occurring oxide layer on the aluminium surface.

When coated with the TMM formulation, the WCA slightly decreased to 75°, indicating a comparable level of wettability to the uncoated surface. Hybrid sol–gel coating maintains moderate water interaction. The alkyl chains in the copolymerised TMM network influence the water droplet shape and WCA, although they do not significantly enhance hydrophobicity.

A substantial increase in hydrophobicity was observed for the TMM coating modified with SiO_2_ + AS, resulting in a WCA of 135°. This nearly circular droplet shape reflected a highly hydrophobic surface, achieved through the improved distribution of SiO_2_ + AS within the TMM matrix compared to its behaviour in THF suspension.

The greatest WCA, at 151°, was recorded for the TMM coating modified with SiO_2_ + FAS. This superhydrophobic surface produced an almost perfectly spherical water droplet, demonstrating minimal interaction with water. The FAS modification further reduced surface energy due to its perfluoro alkyl chains, resulting in a significantly lower wettability than the AS-modified variant. This outcome confirmed that the TMM + SiO_2_ + FAS coating provides the optimal composition for achieving a superhydrophobic aluminium surface.

These results highlighted that while both AS and FAS modifications improve hydrophobicity, the TMM + SiO_2_ + FAS coating is superior in minimising wettability, making it the preferred choice for applications requiring a superhydrophobic surface.

#### 3.2.3. Electrochemical Corrosion Testing

**Figure 12:**
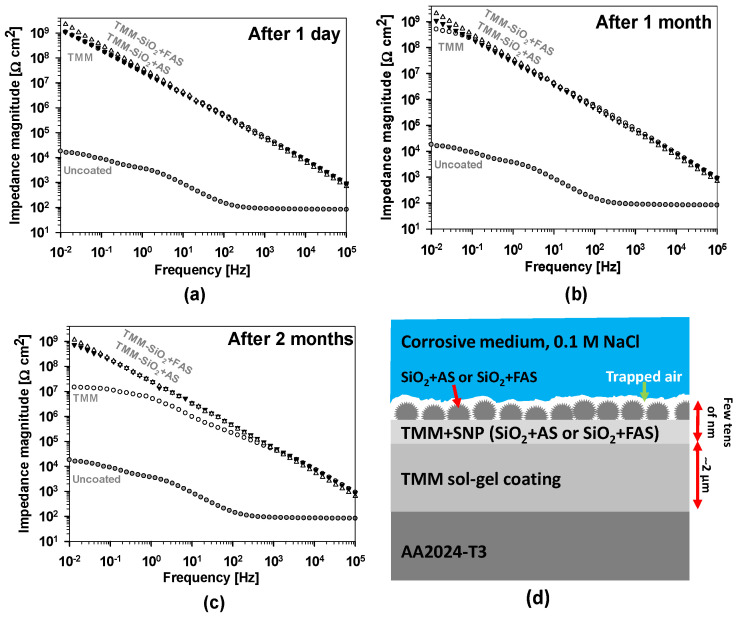
EIS spectra presented as Bode plots of impedance magnitude for AA2024-T3 uncoated and coated with TMM, TMM + SiO_2_ + AS, and TMM + SiO_2_ + FAS coatings recorded at different immersion times: (**a**) one day, (**b**) one month, and (**c**) two months in 0.1 M NaCl. Figure (**d**) presents the mechanism of corrosion protection of the AA2024-T3 coated with a superhydrophobic coating.

The results of the corrosion testing after selected immersion times (1 day, 1 month and 2 months) are presented in Figure 12.

The uncoated AA2024-T3 alloy exhibited a relatively low impedance magnitude at low frequencies of 10 mHz (|*Z*_at 10 mHz_| ≈ 20 kΩ·cm^2^), indicating limited corrosion resistance in the NaCl solution. This behaviour is related to intermetallics Al-Cu-(Fe, Mn) and Al-Cu-Mg [16,17,18,19], representing the area of different electrochemical activity compared to the surrounding matrix covered with Al oxide. To mitigate the galvanic and localised corrosion in chloride medium, additional surface protection is needed. One of the options is the deposition of TMM coatings.

After 1 day, all coated AA2024-T3 samples (TMM, TMM + SiO_2_ + AS, and TMM + SiO_2_ + FAS) demonstrated significantly higher impedance values across the frequency spectrum than the uncoated alloy, highlighting their enhanced corrosion resistance, as shown in Figure 12a. The impedance spectra showed consistent trends across all frequency ranges, with low deviations in the low-frequency region for coated samples. These results validate the reliability and reproducibility of the EIS measurements, ensuring that the observed trends are representative of the coating performance. Great impedance values for coated samples indicate reduced ionic conduction and better barrier properties.

However, only minor variations in impedance response were observed among the TMM, TMM + SiO_2_ + AS, and TMM + SiO_2_ + FAS coatings at low frequencies, shown in Figure 12a. To evaluate the difference between the protective properties of coatings, the vales at |Z| at 10 mHz (|*Z*_at 10 mHz_|) were compared as given in ref. [75]. The TMM + SiO_2_ + FAS coating displayed the highest |*Z*_at 10 mHz_| = 2.2 GΩ cm^2^, compared to that of TMM and TMM + SiO_2_ + AS, |*Z*_at 10 mHz_| = 1.2 GΩ cm^2^, indicating slightly superior corrosion resistance [62,69,70,75]. The superhydrophobic surface created by the FAS-modified SiO_2_ nanoparticles additionally reduces the electrolyte penetration through the TMM coating.

The differences between the coatings are better observed after longer immersion times (1 month and 2 months), shown in Figure 12b,c. After 1 month, the |*Z*_at 10 mHz_| for TMM is reduced 2.2 GΩ cm^2^ and the other two coatings (TMM + SiO_2_ + AS and TMM + SiO_2_ + FAS) remain in the same values. After 2 months, there is a further gradual decrease in |*Z*_at 10 mHz_| for TMM to 15.1 MΩ cm^2^, but three orders of magnitude greater than uncoated 2024-T3 still remain. On the other hand, TMM + SiO_2_ + AS and TMM + SiO_2_ + FAS remain at similar values (|*Z*_at 10 mHz_| = 1.2 GΩ cm^2^ and 0.7 GΩ cm^2^), confirming durable corrosion protection even after 2 months of exposure to the corrosion media.

Figure 12d illustrates the mechanism to explain the EIS results of TMM + SiO_2_ + AS and TMM + SiO_2_ + FAS coatings. The superhydrophobic coating protects the AA2024-T3 substrate from corrosion primarily by forming a highly water-repellent barrier a few tens of nanometres thick. This is achieved by integrating SiO_2_ NPs modified with AS or FAS in a hybrid sol–gel matrix. These NPs, with their low wettability and structured roughness, cause water droplets to form almost spherical shapes, minimising contact with the coating. In addition, air pockets are created at the coating–medium interface, reducing electrolyte penetration and ionic movement.

Additionally, the underlying hybrid sol–gel coating with a thickness of ~2 µm adheres firmly to the AA2024-T3 alloy (as presented by SEM images in Figure 8, Figure 9 and Figure 10), providing a continuous physical barrier that isolates the substrate from direct exposure to moisture and aggressive ions present in the environment (limiting water and ion contact with the substrate), such as chloride ions in NaCl solutions.

In summary, the superhydrophobic coating mechanism on AA2024-T3 involves a dual-layered defence: the sol–gel layer acts as a physical barrier, while the SiO_2_ + FAS layer creates a water-repellent surface that minimises interaction with the corrosive environment. This combination offers an efficient and durable corrosion protection strategy for the aluminium alloy.

#### 3.2.4. Adhesion of the TMM, TMM + SiO_2_ + AS and TMM + SiO_2_ + FAS

**Figure 13:**
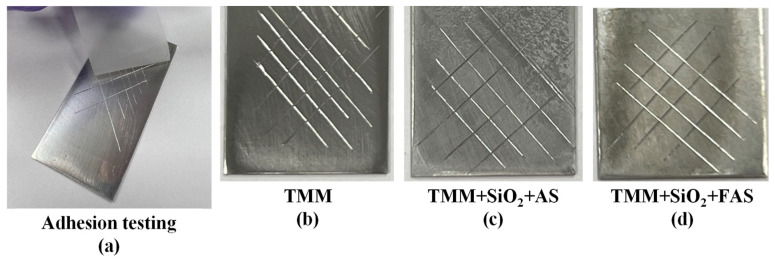
The surface appearance of the AA2024-T3 (**a**) during and after a standard cross-cut test coated with (**b**) TMM, (**c**) TMM + SiO_2_ + AS, and (**d**) TMM + SiO_2_ + FAS coating.

Figure 13 presents the results of the adhesion test performed on AA2024-T3 coated with TMM, TMM + SiO_2_ + AS, and TMM + SiO_2_ + FAS.

In all cases, no peeling or flaking of the coatings was observed along the cross-hatch cuts, indicating that each coating system exhibited strong adhesion to the alloy surface. This excellent adhesion performance suggests that the TMM-based coatings form a stable and durable bond with the substrate, ensuring the coatings remain intact even under mechanical stress.

The strong adhesion of the TMM coating (consisting of siloxane-silica) is attributed to the formation of strong covalent Si–O–Al bonds as presented by Reaction (2) [76,77]:

Al–OH + HO–Si → Si–O–Al + H_2_O (R2)

These bonds form between silanol groups (Si–OH) in the TMM sol–gel network and the hydroxyl groups (Al(OH)_3_) present on the aluminium surface. Future studies could incorporate computational modelling, such as molecular dynamics or density functional theory, to further elucidate the mechanisms of nanoparticle adhesion, wettability, and ion diffusion through the coating.

Incorporating modified SiO_2_ NPs with AS or FAS does not jeopardise the adhesion properties of the TMM coating. TMM + SiO_2_ + AS and TMM + SiO_2_ + FAS coatings adhere firmly to the substrate, benefiting from the underlying covalent bonding mechanism and imparting additional surface properties like enhanced hydrophobicity and long-lasting corrosion protection.

## 4. Conclusions

Our research demonstrated an easy and effective strategy for preparing hydrophobic and superhydrophobic nanoparticles (SiO_2_ + AS and SiO_2_ + FAS) and improving their adhesion to the AA2024-T3 substrate using a hybrid sol–gel coating as a binder.

Based on the obtained results, we have several key findings:

- Nanosized silica SiO_2_ nanoparticles were efficiently synthesised with a size of 25 nm in diameter, which we confirmed by TEM analysis;
- The nanoparticles were successfully surface-modified with AS and FAS molecules (the diameter of NP increased for a few nm). With FAS modification, a more homogeneous distribution of the nanoparticles was achieved compared to AS modification;
- The contact angle measurements proved that FAS with WCA > 150° shows lower wettability than AS with WCA of 134°. The difference is related to the polarity of the alkyl (C−H) and perfluoro alkyl C−F chain in AS and FAS molecules.
- When deposited at the AA2024-T3 by spraying, the film of SiO_2_ nanoparticles did not achieve good adhesion and uniform distribution;
- When the AA2024-T3 was first coated with the TMM hybrid sol–gel coating and then surface-modified using SiO_2_ + AS and SiO_2_ + FAS nanoparticles, the corrosion resistance of AA2024-T3 in 0.1 M NaCl was further enhanced and slightly outperformed the unmodified TMM coating due to the superhydrophobic properties of the coating (trapped air) preventing contact of the surface with the corrosion medium;
- The TMM + SiO_2_ + AS and TMM + SiO_2_ + FAS coatings were corrosion-resistant up to 2 months of exposure to the corrosion medium;
- Cross-cut tests verified that the unmodified TMM coatings and those modified with nanoparticles exhibit high adhesion to the substrate.

This work provides novel directions for robust superhydrophobic coating designs for self-cleaning and corrosion protection applications.

## Figures and Tables

**Table 1:** Results of the EDS analysis of SiO_2_, SiO_2_ + AS, and SiO_2_ + FAS compositions are given in atomic percentage (at.%). EDS was performed on SEM images at a magnification of 50,000× (in Figure 4).

	O	Si	F
SiO_2_	67.7	32.3	/
SiO_2_ + AS	67.1	32.9	/
SiO_2_ + FAS	54.2	32.8	13.0

## Data Availability

Data are available on request.

## Supplementary Materials

The following supporting information can be downloaded at: https://www.mdpi.com/article/10.3390/polym17020195/s1, Figure S1: The FTIR spectra in the provided image appear to compare the transmittance of (i) initial reagent (AS), (ii) SiO_2_ nanoparticles, (iii) SiO_2_ nanoparticles modified with AS (SiO_2_ + AS); Figure S2: The FTIR spectra in the provided image appear to compare the transmittance of (i) initial reagent (FAS), (ii) SiO_2_ nanoparticles, (iii) SiO_2_ nanoparticles modified with FAS (SiO_2_ + FAS); Figure S3: Selected area electron diffraction (SAED) data performed on SiO_2_ nanoparticles; Table S1: Water contact angle measurements were measured on the AA2024-T3, SiO_2_, SiO_2_ + AS, SiO_2_ + FAS, TMM, TMM + SiO_2_ + AS, and TMM + SiO_2_ + FAS surfaces.

## References

[1] Tian X., Verho T., Ras R.H.A. (2016). Moving Superhydrophobic Surfaces toward Real-World Applications. Science.

[2] Vazirinasab E., Jafari R., Momen G. (2018). Application of Superhydrophobic Coatings as a Corrosion Barrier: A Review. Surf. Coat. Technol..

[3] Nakajima A., Hashimoto K., Watanabe T., Takai K., Yamauchi G., Fujishima A. (2000). Transparent Superhydrophobic Thin Films with Self-Cleaning Properties. Langmuir.

[4] Jose A., Gizdavic-Nikolaidis M., Swift S. (2023). Antimicrobial Coatings: Reviewing Options for Healthcare Applications. Appl. Microbiol..

[5] Rodič P., Kapun B., Milošev I. (2022). Superhydrophobic Aluminium Surface to Enhance Corrosion Resistance and Obtain Self-Cleaning and Anti-Icing Ability. Molecules.

[6] Lomga J., Varshney P., Nanda D., Satapathy M., Mohapatra S.S., Kumar A. (2017). Fabrication of Durable and Regenerable Superhydrophobic Coatings with Excellent Self-Cleaning and Anti-Fogging Properties for Aluminium Surfaces. J. Alloys Compd..

[7] Latthe S.S., Sutar R.S., Kodag V.S., Bhosale A.K., Kumar A.M., Kumar Sadasivuni K., Xing R., Liu S. (2019). Self-Cleaning Superhydrophobic Coatings: Potential Industrial Applications. Prog. Org. Coat..

[8] Rodič P., Kovač N., Kralj S., Jereb S., Golobič I., Može M., Milošev I. (2024). Anti-Corrosion and Anti-Icing Properties of Superhydrophobic Laser-Textured Aluminum Surfaces. Surf. Coat. Technol..

[9] Kreder M.J., Alvarenga J., Kim P., Aizenberg J. (2016). Design of Anti-Icing Surfaces: Smooth, Textured or Slippery?. Nat. Rev. Mater..

[10] Rananavare A.P., Jain R., Lee J. (2024). Recent Developments in Superhydrophobic Textiles: A Status Review. J. Coat. Technol. Res..

[11] Bai Y., Zhang H., Shao Y., Zhang H., Zhu J. (2021). Recent Progresses of Superhydrophobic Coatings in Different Application Fields: An Overview. Coatings.

[12] Farag A.A., Mohamed E.A., Toghan A. (2023). The New Trends in Corrosion Control Using Superhydrophobic Surfaces: A Review. Corros. Rev..

[13] Barati Darband G., Aliofkhazraei M., Khorsand S., Sokhanvar S., Kaboli A. (2020). Science and Engineering of Superhydrophobic Surfaces: Review of Corrosion Resistance, Chemical and Mechanical Stability. Arab. J. Chem..

[14] Huda Z., Taib N.I., Zaharinie T. (2009). Characterization of 2024-T3: An Aerospace Aluminum Alloy. Mater. Chem. Phys..

[15] Abreu C.M., Cristóbal M.J., Freitas P., Nóvoa X.R., Pena G., Pérez M.C., Serra C. (2008). Microstructure of the Passive Layer Formed on AA2024-T3 Aluminum Alloy Surface Implanted with Nitrogen. Surf. Interface Anal..

[16] Rodič P., Milošev I., Frankel G.S. (2023). Corrosion of Synthetic Intermetallic Compounds and AA7075-T6 in Dilute Harrison’s Solution and Inhibition by Cerium(III) Salts. J. Electrochem. Soc..

[17] Birbilis N., Buchheit R.G. (2005). Electrochemical Characteristics of Intermetallic Phases in Aluminum Alloys An Experimental Survey and Discussion. J. Electrochem. Soc..

[18] Cavanaugh M.K., Li J.-C., Birbilis N., Buchheit R.G. (2014). Electrochemical Characterization of Intermetallic Phases Common to Aluminum Alloys as a Function of Solution Temperature. J. Electrochem. Soc..

[19] Birbilis N., Buchheit R.G. (2008). Investigation and Discussion of Characteristics for Intermetallic Phases Common to Aluminum Alloys as a Function of Solution pH. J. Electrochem. Soc..

[20] Huang Y., Sarkar D.K., Grant Chen X. (2015). Superhydrophobic Aluminum Alloy Surfaces Prepared by Chemical Etching Process and Their Corrosion Resistance Properties. Appl. Surf. Sci..

[21] Liao R., Zuo Z., Guo C., Yuan Y., Zhuang A. (2014). Fabrication of Superhydrophobic Surface on Aluminum by Continuous Chemical Etching and Its Anti-Icing Property. Appl. Surf. Sci..

[22] Varshney P., Mohapatra S.S., Kumar A. (2016). Superhydrophobic Coatings for Aluminium Surfaces Synthesized by Chemical Etching Process. Int. J. Smart Nano Mater..

[23] Boinovich L.B., Emelyanenko A.M., Modestov A.D., Domantovsky A.G., Emelyanenko K.A. (2015). Synergistic Effect of Superhydrophobicity and Oxidized Layers on Corrosion Resistance of Aluminum Alloy Surface Textured by Nanosecond Laser Treatment. ACS Appl. Mater. Interfaces.

[24] Long J., Zhong M., Zhang H., Fan P. (2015). Superhydrophilicity to Superhydrophobicity Transition of Picosecond Laser Microstructured Aluminum in Ambient Air. J. Colloid Interface Sci..

[25] Volpe A., Gaudiuso C., Di Venere L., Licciulli F., Giordano F., Ancona A. (2020). Direct Femtosecond Laser Fabrication of Superhydrophobic Aluminum Alloy Surfaces with Anti-Icing Properties. Coatings.

[26] Može M., Senegačnik M., Gregorčič P., Hočevar M., Zupančič M., Golobič I. (2020). Laser-Engineered Microcavity Surfaces with a Nanoscale Superhydrophobic Coating for Extreme Boiling Performance. ACS Appl. Mater. Interfaces.

[27] Park B.G., Lee W., Kim J.S., Lee K.B. (2010). Superhydrophobic Fabrication of Anodic Aluminum Oxide with Durable and Pitch-Controlled Nanostructure. Colloids Surf. Physicochem. Eng. Asp..

[28] Saji V.S. (2020). Superhydrophobic Surfaces and Coatings by Electrochemical Anodic Oxidation and Plasma Electrolytic Oxidation. Adv. Colloid Interface Sci..

[29] Li W., Zhan Y., Yu S. (2021). Applications of Superhydrophobic Coatings in Anti-Icing: Theory, Mechanisms, Impact Factors, Challenges and Perspectives. Prog. Org. Coat..

[30] Shen X., Mao T., Li C., Mao F., Xue Z., Xu G., Amirfazli A. (2023). Durable Superhydrophobic Coatings Based on CNTs-SiO2gel Hybrids for Anti-Corrosion and Thermal Insulation. Prog. Org. Coat..

[31] Wang H., Chen E., Jia X., Liang L., Wang Q. (2015). Superhydrophobic Coatings Fabricated with Polytetrafluoroethylene and SiO2 Nanoparticles by Spraying Process on Carbon Steel Surfaces. Appl. Surf. Sci..

[32] Zhang X.-F., Li X.-D., Wang N., Liu Y.-J., Tian F., Wang C.-X. (2023). Robust Superhydrophobic SiO_2_/Epoxy Composite Coating Prepared by One-Step Spraying Method for Corrosion Protection of Aluminum Alloy: Experimental and Theoretical Studies. Mater. Des..

[33] Alexander S., Eastoe J., Lord A.M., Guittard F., Barron A.R. (2016). Branched Hydrocarbon Low Surface Energy Materials for Superhydrophobic Nanoparticle Derived Surfaces. ACS Appl. Mater. Interfaces.

[34] Kumar A., Gogoi B. (2018). Development of Durable Self-Cleaning Superhydrophobic Coatings for Aluminium Surfaces via Chemical Etching Method. Tribol. Int..

[35] Varshney P., Lomga J., Gupta P.K., Mohapatra S.S., Kumar A. (2018). Durable and Regenerable Superhydrophobic Coatings for Aluminium Surfaces with Excellent Self-Cleaning and Anti-Fogging Properties. Tribol. Int..

[36] Sun R., Zhao J., Li Z., Mo J., Pan Y., Luo D. (2019). Preparation of Mechanically Durable Superhydrophobic Aluminum Surface by Sandblasting and Chemical Modification. Prog. Org. Coat..

[37] Yang J., Li J., Xu P., Chen B. (2020). Robust and Transparent Superoleophobic Coatings from One-Step Spraying of SiO2@fluoroPOS. J. Sol-Gel Sci. Technol..

[38] Cheng Q.-Y., An X.-P., Li Y.-D., Huang C.-L., Zeng J.-B. (2017). Sustainable and Biodegradable Superhydrophobic Coating from Epoxidized Soybean Oil and ZnO Nanoparticles on Cellulosic Substrates for Efficient Oil/Water Separation. ACS Sustain. Chem. Eng..

[39] Qiu X., Wang Y. (2023). Preparation of Superhydrophobic Composite Coating on 2024 Aluminum Alloy and Its Stability and Corrosion Resistance. Int. J. Electrochem. Sci..

[40] Du Y., Hu L., Dong L., Du S., Xu D. (2023). Experimental Study on Anti-Icing of Robust TiO2/Polyurea Superhydrophobic Coating. Coatings.

[41] Zhang H.H., Zhang X., Bian H., Zhang L., Chen Y., Yang Y., Zhang Z. (2024). Superhydrophobic and Self-Healing Silane-Ceria Composite Coating Implanted with 1,5-Naphthalenediol Inhibitor for Corrosion Protection of AA2024-T3 Aluminum Alloy. Surf. Coat. Technol..

[42] Park J.T., Seo J.A., Ahn S.H., Kim J.H., Kang S.W. (2010). Surface Modification of Silica Nanoparticles with Hydrophilic Polymers. J. Ind. Eng. Chem..

[43] Jung H.-S., Moon D.-S., Lee J.-K. (2012). Quantitative Analysis and Efficient Surface Modification of Silica Nanoparticles. J. Nanomater..

[44] Ammar S., Ramesh K., Ma I.A.W., Farah Z., Vengadaesvaran B., Ramesh S., Arof A.K. (2017). Studies on SiO2-Hybrid Polymeric Nanocomposite Coatings with Superior Corrosion Protection and Hydrophobicity. Surf. Coat. Technol..

[45] Chen H., Zhang X., Zhang P., Zhang Z. (2012). Facile Approach in Fabricating Superhydrophobic SiO2/Polymer Nanocomposite Coating. Appl. Surf. Sci..

[46] Ghodrati M., Mousavi-Kamazani M., Bahrami Z. (2023). Synthesis of Superhydrophobic Coatings Based on Silica Nanostructure Modified with Organosilane Compounds by Sol–Gel Method for Glass Surfaces. Sci. Rep..

[47] Qiu X., Li J., Wang J., Yang X., Li Y., Qi D. (2022). A Robust Superhydrophobic and Oleophobic Coating with Short Chain Perfluoroalkyl Group and Flower-Shaped SiO_2_ Nanoparticles. Surf. Coat. Technol..

[48] Devaprakasam D., Sampath S., Biswas S.K. (2004). Thermal Stability of Perfluoroalkyl Silane Self-Assembled on a Polycrystalline Aluminum Surface. Langmuir.

[49] Rodič P., Kapun B., Panjan M., Milošev I. (2020). Easy and Fast Fabrication of Self-Cleaning and Anti-Icing Perfluoroalkyl Silane Film on Aluminium. Coatings.

[50] Liu H., Huang J., Chen Z., Chen G., Zhang K.-Q., Al-Deyab S.S., Lai Y. (2017). Robust Translucent Superhydrophobic PDMS/PMMA Film by Facile One-Step Spray for Self-Cleaning and Efficient Emulsion Separation. Chem. Eng. J..

[51] Gu H., Zhang Q., Gu J., Li N., Xiong J. (2018). Facile Preparation of Superhydrophobic Silica Nanoparticles by Hydrothermal-Assisted Sol–Gel Process and Effects of Hydrothermal Time on Surface Modification. J. Sol-Gel Sci. Technol..

[52] Harb S.V., Cerrutti B.M., Pulcinelli S.H., Santilli C.V., Hammer P. (2015). Siloxane–PMMA Hybrid Anti-Corrosion Coatings Reinforced by Lignin. Surf. Coat. Technol..

[53] Harb S.V., dos Santos F.C., Caetano B.L., Pulcinelli S.H., Santilli C.V., Hammer P. (2015). Structural Properties of Cerium Doped Siloxane–PMMA Hybrid Coatings with High Anticorrosive Performance. RSC Adv..

[54] Harb S.V., Trentin A., de Souza T.A.C., Magnani M., Pulcinelli S.H., Santilli C.V., Hammer P. (2020). Effective Corrosion Protection by Eco-Friendly Self-Healing PMMA-Cerium Oxide Coatings. Chem. Eng. J..

[55] Judeinstein P., Sanchez C. (1996). Hybrid Organic-Inorganic Materials: A Land of Multidisciplinarity. J. Mater. Chem..

[56] Harb S.V., Trentin A., Torrico R.F.O., Pulcinelli S.H., Santilli C.V., Hammer P., Giudice C., Canosa G. (2017). Organic-Inorganic Hybrid Coatings for Corrosion Protection of Metallic Surfaces. New Technologies in Protective Coatings.

[57] Ali U., Karim K.J.B.A., Buang N.A. (2015). A Review of the Properties and Applications of Poly (Methyl Methacrylate) (PMMA). Polym. Rev..

[58] Sanchez C., Julián B., Belleville P., Popall M. (2005). Applications of Hybrid Organic–Inorganic Nanocomposites. J. Mater. Chem..

[59] Abdolah Z.M., van der Sybrand Z., Garcia S.J. (2013). Routes to Extrinsic and Intrinsic Self-Healing Corrosion Protective Sol-Gel Coatings: A Review. Self-Heal. Mater..

[60] Figueira R.B. (2020). Hybrid Sol–Gel Coatings for Corrosion Mitigation: A Critical Review. Polymers.

[61] Rodič P., Mertelj A., Borovšak M., Benčan A., Mihailović D., Malič B., Milošev I. (2016). Composition, Structure and Morphology of Hybrid Acrylate-Based Sol–Gel Coatings Containing Si and Zr Composed for Protective Applications. Surf. Coat. Technol..

[62] Hamulić D., Rodič P., Poberžnik M., Jereb M., Kovač J., Milošev I. (2020). The Effect of the Methyl and Ethyl Group of the Acrylate Precursor in Hybrid Silane Coatings Used for Corrosion Protection of Aluminium Alloy 7075-T6. Coatings.

[63] Rodič P., Iskra J., Milošev I. (2014). A Hybrid Organic–Inorganic Sol–Gel Coating for Protecting Aluminium Alloy 7075-T6 against Corrosion in Harrison’s Solution. J. Sol-Gel Sci. Technol..

[64] Rodič P., Lekka M., Andreatta F., Fedrizzi L., Milošev I. (2020). The Effect of Copolymerisation on the Performance of Acrylate-Based Hybrid Sol-Gel Coating for Corrosion Protection of AA2024-T3. Prog. Org. Coat..

[65] Trentin A., Gasparini A.d.L., Faria F.A., Harb S.V., dos Santos F.C., Pulcinelli S.H., Santilli C.V., Hammer P. (2020). Barrier Properties of High Performance PMMA-Silica Anticorrosion Coatings. Prog. Org. Coat..

[66] Milošev I., Hamulić D., Rodič P., Carrière C., Zanna S., Budasheva H., Korte D., Franko M., Mercier D., Seyeux A. (2022). Siloxane Polyacrylic Sol-Gel Coatings with Alkly and Perfluoroalkyl Chains: Synthesis, Composition, Thermal Properties and Log-Term Corrosion Protection. Appl. Surf. Sci..

[67] Uvida M.C., Trentin A., Harb S.V., Pulcinelli S.H., Santilli C.V., Hammer P. (2022). Nanostructured Poly(Methyl Methacrylate)–Silica Coatings for Corrosion Protection of Reinforcing Steel. ACS Appl. Nano Mater..

[68] Pan S., Wang N., Xiong D., Deng Y., Shi Y. (2016). Fabrication of Superhydrophobic Coating via Spraying Method and Its Applications in Anti-Icing and Anti-Corrosion. Appl. Surf. Sci..

[69] Rodič P., Korošec R.C., Kapun B., Mertelj A., Milošev I. (2020). Acrylate-Based Hybrid Sol-Gel Coating for Corrosion Protection of AA7075-T6 in Aircraft Applications: The Effect of Copolymerization Time. Polymers.

[70] Hamulić D., Medoš G., Korte D., Rodič P., Milošev I. (2023). The Effect of Curing Temperature and Thickness of Polybutyl Methacrylate Siloxane Coatings on the Corrosion Protection of Structural Steel S355. Coatings.

[71] ASTM International (2022). ASTM D7334-08(2022) Standard Guide for the Evaluation of the Performance of Protective Clothing and Equipment Against Chemical Agents.

[72] Milošev I., Bakarič T., Zanna S., Seyeux A., Rodič P., Poberžnik M., Chiter F., Cornette P., Costa D., Kokalj A. (2019). Electrochemical, Surface-Analytical, and Computational DFT Study of Alkaline Etched Aluminum Modified by Carboxylic Acids for Corrosion Protection and Hydrophobicity. J. Electrochem. Soc..

[73] Poberžnik M., Chiter F., Milošev I., Marcus P., Costa D., Kokalj A. (2020). DFT Study of *n*-Alkyl Carboxylic Acids on Oxidized Aluminum Surfaces: From Standalone Molecules to Self-Assembled-Monolayers. Appl. Surf. Sci..

[74] dos Santos F.C., Pulcinelli S.H., Santilli C.V., Hammer P. (2021). Protective PMMA-Silica Coatings for Aluminum Alloys: Nanostructural Control of Elevated Thermal Stability and Anticorrosive Performance. Prog. Org. Coat..

[75] Cristoforetti A., Rossi S., Deflorian F., Fedel M. (2023). On the Limits of the EIS Low-Frequency Impedance Modulus as a Tool to Describe the Protection Properties of Organic Coatings Exposed to Accelerated Aging Tests. Coatings.

[76] Poberžnik M., Costa D., Hemeryck A., Kokalj A. (2018). Insight into the Bonding of Silanols to Oxidized Aluminum Surfaces. J. Phys. Chem. C.

[77] Poberžnik M., Kokalj A. (2019). Implausibility of Bidentate Bonding of the Silanol Headgroup to Oxidized Aluminum Surfaces. Appl. Surf. Sci..

