# Supplementary material for: Superhydrophobic Coatings Based on PMMA-Siloxane-Silica and Modified Silica Nanoparticles Deposited on AA2024-T3
Source: Polymers (Basel). 2025 Jan 14;17(2):195. doi: 10.3390/polym17020195 (PMC11769079; doi:10.3390/polym17020195)
Supplement: Supplementary file 1 [file polymers-17-00195-s001.zip › polymers-3374427-supplementary.pdf]

polymers

MDPI

Superhydrophobic Coatings Based on PMMA-Siloxane-Silica and Modified Silica Nanoparticles Deposited on AA2024-T3

Nina Kovač[1,2], Barbara Kapun[1], Matic Može[3], Iztok Golobič[3], Slavko Kralj[4,5], Ingrid Milošev[1], Peter Rodič[1,*]

[1] Jožef Stefan Institute, Department of Physical and Organic Chemistry, Jamova c. 39, SI-1000 Ljubljana, Slovenia; nina.kovac@ijs.si (N.K.); barbara.kapun@ijs.si (B.K.); ingrid.milosev@ijs.si (I.M.)
[2] Jožef Stefan International Postgraduate School, SI-1000 Ljubljana, Slovenia
[3] Faculty of Mechanical Engineering, University of Ljubljana, Aškerčeva c. 6, SI-1000 Ljubljana, Slovenia; matic.moze@fs.uni-lj.si (M.M.); iztok.golobic@fs.uni-lj.si (I.G.)
[4] Jožef Stefan Institute, Department for Materials Synthesis, Jamova c. 39, SI-1000 Ljubljana, Slovenia; slavko.kralj@ijs.si
[5] Faculty of Pharmacy, University of Ljubljana, Aškerčeva c. 7, SI-1000 Ljubljana, Slovenia
* Corresponding author; e-mail: peter.rodic@ijs.si (P. Rodič); Phone.: + 386 1 4773 261

SUPPLEMENT MATERIAL

1.1 FTIR spectra

Figures S1 and S2 present the FTIR spectra performed using the spectrometer ALPHA II with the diamond ATR module. The spectra were analysed using OPUS software.

In Figure S1a, the AS solution (as a reference) has several characteristic bands in the FTIR spectra, such as C–H Stretch (~2800–3000 cm^{-1} and (~1350–1500 cm^{-1}) and Si–OCH$_x$ bands at (1078, 950 and 780 cm^{-1}). SiO_2 and SiO_2+AS show additional bands, indicating the presence of SiO_2 formation and surface functionalization. The broad bands ~3500 cm^{-1} to ~3000 cm^{-1} are likely associated with hydroxyl groups O-H in the recorded spectra.

C–H$_x$ stretches (~2800–3000 cm^{-1}) are present in the AS and SiO_2+AS but absent in SiO_2, suggesting organic groups in AS (partly related to alkoxy groups, but mainly related to alkyl chain in the AS molecule). This confirmed that AS was successfully grafted onto SiO_2.

The zoomed area in the wavenumber range (~1600 cm^{-1} to ~500 cm^{-1}) (Figure S1b) showed Si–O–Si tretch (~1050 cm^{-1} and at 800 cm^{-1} associated with the asymmetric and symmetric Si–O–Si stretching vibration bonding) which are characteristic strong band for silica observed in SiO_2 and SiO_2+AS (these are seen shifts compared to bands present in the AS). Band (1078, 950 cm^{-1} and 780 cm^{-1}) in AS diminishes in SiO_2–AS, indicating successful modification. A band related to Si–OH (~940 cm^{-1}) is present due to the presence of free hydroxyl groups (Si–OH). AS bands (~1400–1500 cm^{-1}) also appear in SiO_2-AS but not unmodified SiO_2, confirming AS's presence.

Similar behaviour was also noticed for FAS, SiO_2 and SiO_2+FAS. The main difference is less intensive bands related to C–H Stretch (~2800–3000 cm^{-1}, mainly related to alkoxy groups), and more intensive bands (~1150–1250 cm^{-1}) related to C–F bands.

The FTIR confirmed that the spectral differences between SiO_2 and SiO_2+AS/ SiO_2+FAS indicate successful grafting of AS/FAS onto the SiO_2 nanoparticles. A decrease in Si–OH bands in SiO_2+AS/ SiO_2+FAS suggests that the surface hydroxyl groups of SiO_2 reacted with AS/FAS during modification.

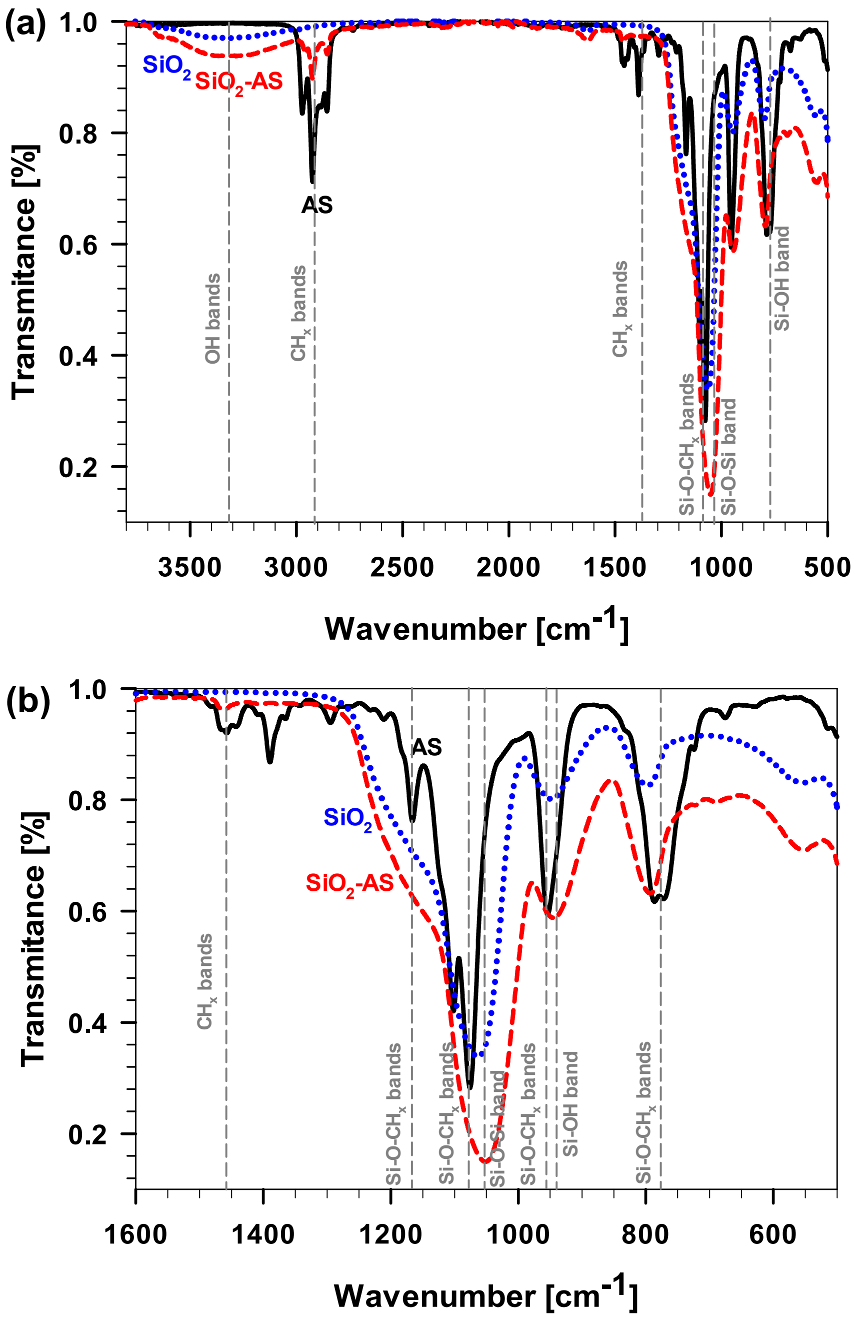

Figure S1. The FTIR spectra in the provided image appear to compare the transmittance of (i) initial reagent (AS), (ii) SiO_2 nanoparticles, (iii) SiO_2 nanoparticles modified with AS (SiO_2+AS).

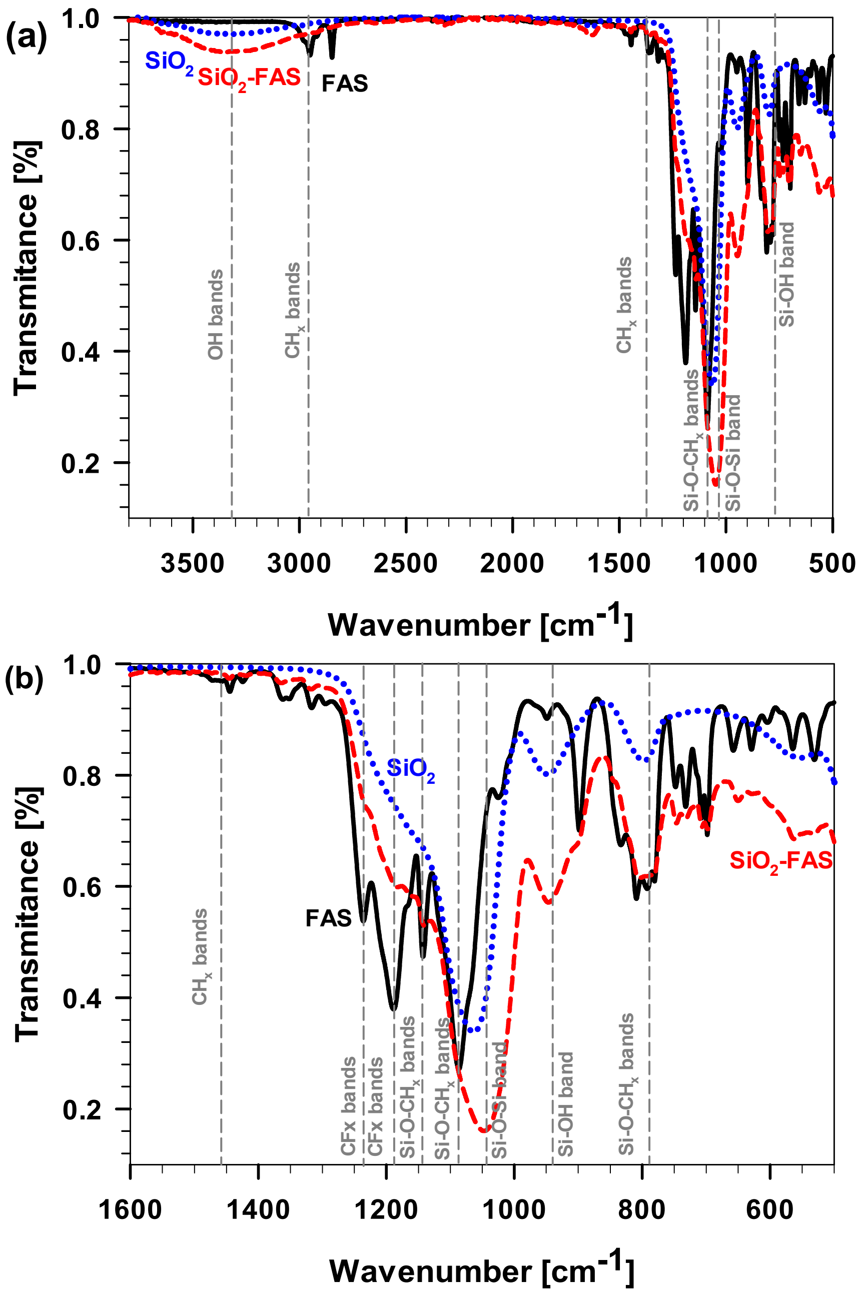

Figure S2. The FTIR spectra in the provided image appear to compare the transmittance of (i) initial reagent (FAS), (ii) SiO_2 nanoparticles, (iii) SiO_2 nanoparticles modified with FAS (SiO_2+FAS).

1.2 Selected area electron diffraction (SAED) data

Selected area electron diffraction (SAED) analysis confirmed the amorphous structure of the SiO_2 nanoparticles. This aligns with the Stöber synthesis process, where amorphous structure is expected.

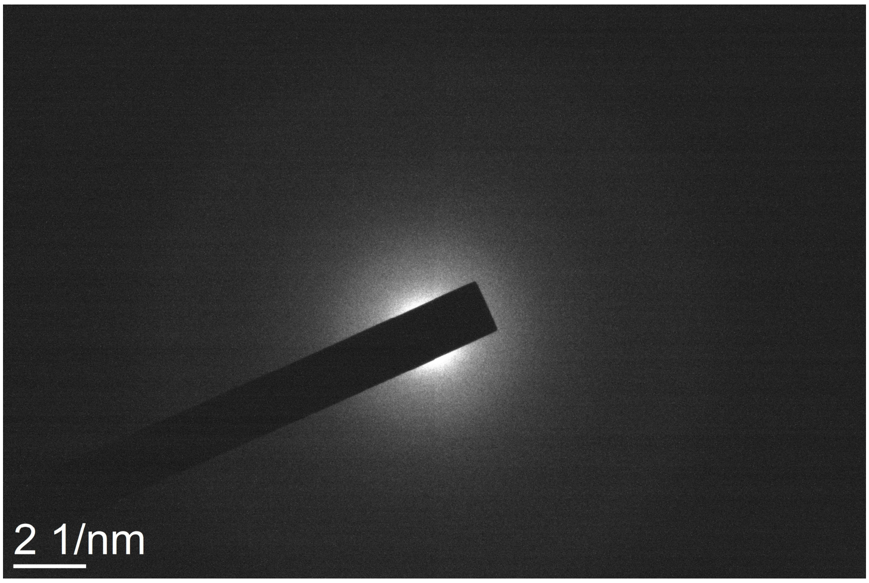

Figure S3. Selected area electron diffraction (SAED) data performed on SiO_2 nanoparticles.

1.3 Water contact angle measurements

Table S1. Water contact angle measurements were measured on the AA2024-T3, SiO_2, SiO_2+AS, SiO_2+FAS, TMM, TMM+SiO_2+AS, and TMM+SiO_2+FAS surfaces.

**Sample name/number of measurement**	**Contact angle [°]**
AA2024-T3	
0-0	79.7
0-1	87.1
0-2	79.2
Average + standard deviation	82.0 ± 4.44
SiO_2	
0-0	65.2
0-1	77.6
0-2	70.8
Average + standard deviation	71.2 ± 6.19
SiO_2+AS	
0-0	94.8
0-1	85.2
0-2	106.9
Average + standard deviation	95.7 ± 10.86
SiO_2+FAS	
0-0	154.2
0-1	153.5
0-2	155.6
Average + standard deviation	154.4 ± 0.87
TMM	
0-0	74.6
0-1	75.2
0-2	75.9
Average + standard deviation	75.2 ± 0.53
TMM+SiO_2+AS	
0-0	133.7
0-1	137.3
0-2	135.2
Average + standard deviation	135.4 ± 1.47
TMM+SiO_2+FAS	
0-0	151.3
0-1	152.5
0-2	150.1
Average + standard deviation	151.3 ± 1.00
